# The potential of intervention-based community development programs in reducing multiple health risk behaviors among adolescent: A scoping review of the latest RCTs

**DOI:** 10.34172/hpp.2022.20

**Published:** 2022-08-20

**Authors:** Ahmad Yamin, Suryani Suryani, Siti Yuyun Rahayu, Neti Juniarti

**Affiliations:** ^1^Department of Community Health Nursing, Faculty of Nursing, Universitas Padjadjaran, Bandung, Indonesia; ^2^Department of Mental Health Nursing, Faculty of Nursing, Universitas Padjadjaran, Bandung, Indonesia; ^3^Department of Pediatric Nursing, Faculty of Nursing, Universitas Padjadjaran, Bandung, Indonesia; ^4^Department of Community Health Nursing, and Continuity of Care Research Center, Faculty of Nursing, Universitas Padjadjaran, Bandung, Indonesia

**Keywords:** Adolescent, Community development/social plannings, Health risk behaviors

## Abstract

**Background:** Adolescents are more likely than adults to engage in risky health behaviors such as smoking, drinking, and sexual activity. Community development plays a role in reducing adolescents’ personal, cognitive, and social skill deficits. A review of the effectiveness of community-development interventions is required to advance our understanding of how the intervention reduce health risk behaviors. This study analyze type and effectiveness of adolescents’ community development programs reduce multiple health risk behaviors among adolescents.

**Methods:** This scoping review used the Preferred Reporting Items for Systematic Reviews and Meta-Analyses Extension for Scoping Reviews (PRISMA-ScR). With a date range of 2015–2021, searches were conducted in PubMed, EBSCO, and ProQuest using keywords (((Life skill education) OR (community development)) AND ((health risk behavior) OR (risk behavior)) AND ((adolescent) OR (adolescence) OR (teenagers) OR (teens) OR (youth))). After title and abstract checking, full-text retrieval, and data extraction, data were synthesized based on the main objectives. The most important data were tabulated.

**Results:** Most studies showed that community development–based interventions effectively reduce adolescents’ health risk behaviors, including risky sexual behaviors, drug and alcohol use. Interventions were carried out in schools, places of worship, and communities, involving adolescents, educational institutions, health professionals, religious leaders, and families.

**Conclusion:** This review can assist community health nurses, policymakers, researchers, and teachers in developing and implementing effective community-development programs that ensure knowledge, attitudes, and skills transfer to reduce health risk behaviors.

## Introduction

 Adolescence is a period of fast development and growth, both physically and psychologically. Adolescents show high interest in exploring new things, and trying new risky experiences, regardless the implication of their action.^1^ As young people have less control over their emotions, it is easier for them to engage in unhealthy behaviors and/or make poor lifestyle choices.^2^

 Adolescence is related with an increased prevalence of health-risking behaviors such as drugs abuse, sexual risk-taking, and violence actions.^3^ Most substance abuse begins during adolescence. Many adolescents have been smoking since elementary school, if not earlier. In terms of alcohol consumption among youth, 3.3% start drinking before 10 years old.^1^ Adolescence also have associated with higher rates of sexually transmitted disease and abortion. Since 2014, sexually transmitted infection rates in adolescents have risen, with young women and men who have sex with men being especially vulnerable.^4^ Unintentional injury is the leading cause of morbidity and mortality among adolescents,^5,6^ with motor vehicle accidents the predominant cause of those injuries. Drowning, poisoning, and fire/burns are also other common causes of unintentional injuries.^6^

 Emotional, cognitive, behavioral, and resilience skills are critical to adolescents’ personal and social success. These skills can be used as resources to overcome challenges in adolescents’ life as well as to contribute to the community.^7^ Community development plays a role in reducing adolescents’ personal, cognitive, and social skill deficits. Community development is defined as “a process of developing and enhancing the ability to act collectively.”^7^ In this study, “community development-based intervention” is defined as the intervention of developing and enhancing the ability to act collectively in reducing multiple health risk behaviors among adolescents. Adolescent health needs support from families and peers in school, also from their social environment at a population level.^8^ Alcohol and drugs abuse; sexual abuse, mental health problems, and disturbance of physical health; and lower occupational and educational level are related to adolescents’ early health risk behaviors.^9^ A recent scoping review shows the strongest empirical support for school-based, mentoring and wilderness, adventure, and outdoor programming formats to improve healthy adolescent development.^10^ However, methodological concerns were identified in the review, and all the interventions focused on individual adolescents and lacked intervention to enhance the community’s ability to collaborate in reducing multiple health risk behaviors among adolescents.

 A review of the effectiveness of community-development interventions is required to advance our understanding of what and how community development interventions reduce health risk behaviors. This study aimed to analyze type and effectiveness of adolescents’ community development programs reduce multiple health risk behaviors among adolescents.

## Material and Methods

###  Study design

 This scoping review was carried out following the Joanna Briggs Institute methodology,using the framework developed by Arksey & O’Malley^11^ and the Preferred Reporting Items for Systematic Reviews and Meta-Analyses Extension for Scoping Reviews (PRISMA-ScR).^10^ The scoping review started with a research questions that was narrowed throughout the study to allow for a more extensive evaluation of outcomes.^11^

###  Search methods

 A thorough search strategy was implemented to locate published literature in English from 2015 to November 2021. Peters et al^11^ recommended for scoping reviews used only studies published in English were considered. PubMed, EBSCO, and ProQuest were among the databases used to find published literature. A Medical Subject Heading (MeSH)–adjusted literature search was conducted using keywords and the following Boolean operators: (((life skill education) OR (community development)) AND ((health risk behavior) OR (risk behavior)) AND ((adolescent) OR (adolescence) OR (teenagers) OR (teens) OR (youth))).

###  Eligibility criteria

 Inclusion criteria for eligible articles were including:

1. Population: adolescents as participants/samples
2. Concept: Study is about community-development using randomized controlled trial (RCT) designs were included in the concept (study designs)
3. Context: Study focuses on reducing health risk behaviors

###  Data collection and analysis

 After data collection and study selection process, authors identified studies using the PRISMA flow chart: (1) identification and exclusion of duplicates, (2) screen articles’ titles and abstracts (3) articles with full-text availability were included. the studies were identified following the PRISMA flow chart: (1) duplicates were identified and excluded, (2) titles and abstracts were screened, and (3) articles with full-text availability were included. The data were extracted from the study results manually and then tabulated based on author, country, study design, intervention model, and effectiveness were among the data items searched. A thematic analysis method was used to examine the data.

## Results

###  Selection

**Figure 1:**
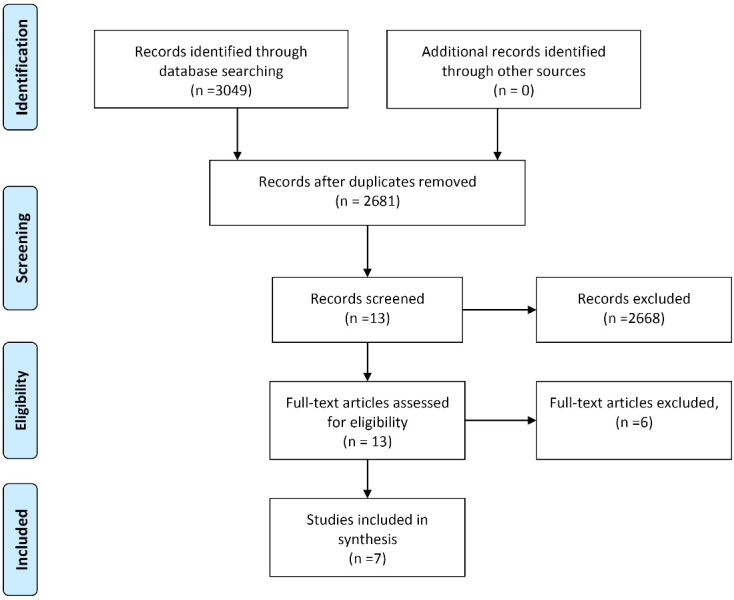
PRISMA-ScR flow chart

 The literature search yielded 2729 articles, of which 2681 remained after duplicate removal. From the 13 full texts evaluated for eligibility, seven articles^12-21^ were included in the systemic review (Figure 1).

###  Study characteristics

 Participants in the pooled studies had various demographic characteristics and average ages (Table 1). The studies were conducted in the United States (n = 2), Brazil (n = 2), Kenya (n = 2), the Pacific Northwest (n = 1), and the Netherlands (n = 1). All studies had a randomized controlled trial (RCT) design and were conducted on approximately 7916 adolescents; however, one study did not report the sample size.

**Table 1:** Characteristics of selected studies

**Country**	**Study design **	**Population**	**Sample (n)**	**Age (y)**	**Male (%)**	**Reference**
USA	Cluster RCT	Latina adolescents (students)	NI	NI	NI	^19^
Brazil	RCT	Students in 72 public schools spread across six Brazilian cities	6,391	12.6 ± 0.8	49	^17^
Kenya	Longitudinal randomized controlled effectiveness trial	Students	182	NI	NI	^14^
Kenya	RCT	Adolescents	237	12.3 ± 2.0	48.1	^15^
USA	Randomized feasibility study	Adolescents	162	Intervention: 16.8 Control: 16.9	Intervention: 50.63 Control: 56.99	^18^
Pacific Northwest	RCT	Early adolescent girls in foster care	145	11.54 ± 0.48	0	^20^
Netherlands	Cluster RCT	Adolescents in secondary schools	699	13–15	Intervention: 47 Control: 57	^21^

NI, no information; RCT, randomized controlled trial; USA, the United States.

###  Intervention modalities

 Several community development empowerment–based intervention modalities were found. The interventions involved schools, places of worship, and the community. Most of the interventions were integrated with youth activities at school. Interventions are carried out in the school environment because teenagers are of school age. Many adolescent activities are carried out in the school environment (see Table 2).

**Table 2:** Summary of findings

**Reference**	**Intervention**	**Health risk behavior**	**Efficacy **
19	Mobile-phone contraception-decision support intervention	Pregnancy in adolescents	Based on user input, the app offers tailored contraceptive recommendations and asks young people which methods they are most interested in. Before the in-person visit, this information is shared with the provider
17	School-based drug-prevention program	Sexual behavior, alcohol, nicotine, and other illicit narcotics, and teen dating violence	At the 21-month follow-up, receipt of the program was associated with a higher risk of lifetime sex among all participants (OR = 1.27, 95%CI 1.03 - 1.56).
14	HIV prevention program	HIV prevention	The slope coefficients for four variables, based on multilevel models, showed a reliable change in the desired direction: abstinence from oral, vaginal, or anal sex in the previous two months; condom attitudes; HIV testing; and refusal skills
15	Church- and family-based	Mental health problems and HIV prevention	In comparison to controls, the intervention group reported better family communication across domains at one and three months post-intervention (*P* < 0.001), as well as higher self-efficacy (*P* < 0.001) for risk-reduction skills and HIV-related knowledge (*P* < 0.1) at one month. One month post-intervention, sexually active adolescents reported fewer high-risk behaviors, such as unprotected sex or multiple partners (*P* < 0.1)
18	Conditional cash transfer and intervention in life skills	Health-risking sexual and drinking	There is no evidence that cash payments were used to fund illegal or high-risk behavior. At six months, intervention participants had a lower likelihood of frequently health risking sexual and drinking (OR = 0.54, *P* = 0.10) and a lower likelihood of reporting that close friends had been incarcerated (OR = 0.6, *P* = 0.12). They consumed less alcohol on a regular basis (OR = 0.54, *P* = 0.04) and had fewer sexual encounters (OR = 0.50, *P* = 0.04)
20	MSS intervention	Sexual behavior	Adolescents in the intervention group, reported 89 high risk sexual behavior (SD = 1.17), while adolescent in the comparison group reported 1.69 acts of high-risk behavior (SD = 2.04)
21	Selective school-based alcohol-prevention program	Drinking	At 12 months post intervention, there was no significant difference in binge drinking rates between the intervention and control groups (OR = 1.05; 95% CI 0.99 – 1.11), and intention-to-treat analyses revealed no significant intervention effects on alcohol use (OR = 0.99; 95% CI 0.86, - 1.14) or problem drinking (OR = 1.03; 95% CI 0.92 -1.10) Post-hoc latent-growth analyses revealed significant effects on binge drinking development (β = –0.16, *P* = 0.05) and frequency *( β* = –0.14, *P* = 0.05)

CI, confidence interval; MSS, Middle School Success; OR, odds ratio; SD, standard deviation.

###  School environment

 Interventions carried out in the school environment were included a mobile-phone contraception-decision support intervention,^20^ a school-based drug-prevention program,^18^ the Middle School Success (MSS) intervention,^21^ and a selective school-based alcohol-prevention program.^22^A “mobile-phone contraception-decision support intervention” is a mobile-phone application that offers interactive sexual-health information and contraception-decision support. The clinician received a printout from the app, and then the adolescent meet face to face with the clinician.^19^

 The school-based drug-prevention intervention provided 12 sessions, one per week. Trained teachers led the twelve 50-minute classroom sessions. The intervention of 12 sessions was a modified version of the *Unplugged* show that employed a social-influence strategy to address social and personal skills, knowledge, and normative beliefs. The intervention focus on three broad areas drugs abuse, interpersonal skills and intrapersonal skills.^17^

 The MSS was a program to prevent health-risking sexual behavior that consists of two parts, the first part was session for parents and the second part was for adolescents for duration of three weeks, twice activities a week followed by an ongoing training and support once a week for two hours.^20^

 Finally, the selective school-based alcohol-prevention program to prevent drinking behavior consisted of two group sessions of six student with duration of 90 minutes over two weeks held at the participants’ schools during school hours in the Netherlands. Student manuals were used in the intervention. The program manuals’ text, examples, exercises, real-life stories or scenarios, and illustrations were tailored to the Dutch context. Previously organized focus groups of adolescents’ role-played scenarios with high-risk personalities. Psycho-educational strategies were used in the first group. Students were motivated to explore their personalities and ways of coping with their characters through practicing a goal-setting activity. The cognitive-behavioral model was then introduced. In the second session, participants were encouraged to identify and challenge problematic behaviors that caused by personality-specific cognitive thoughts such as the impulsivity intervention and the sensation-seeking intervention.^21^

###  Place of worship

 The modality used in a place of worship was a church- and family-based intervention.^15^ This intervention started with a 60-minute family session focused on in-session communication practice. After that, adolescents met in gender-segregated groups for another 60 minutes of discussion and skill practice. Concurrently, families discussed application on daily life for 30 minutes before breaking into male and female discussion groups for the final 30 minutes. The intervention was followed up by a weekly discussion groups for church leaders to identify ways and further intervention for their congregation that will be given at the last intervention session.^15^

###  Community setting

 The modalities in the community setting were an HIV prevention program^14^ and a conditional cash transfer and intervention in life skills.^18^ The first HIV prevention program created a peer educator training intervention for university students. The curriculum focused on “risk elimination and included a variety of risk-reduction strategies based on the ABC (abstinence from sexual activity, being faithful to a single partner, and correct and consistent condom use) approach to HIV prevention”.^14^ The conditional cash transfer and intervention in life skills lasted six months, with an eight-session life skills group offered weekly for the first two months. This intervention identified 24 participation and performance goals related to postsecondary education, job training, and reproductive health wellness. Adolescents received small cash payments contingent after completing the given tasks. This intervention encouraged participants to set objectives corresponding to their needs. The life skills sessions improve sexual health, focusing on sexually transmitted disease and unintended pregnancy prevention, and early childbearing norms.^18^

###  Outcomes of multiple health risk behaviors

**Figure 2:**
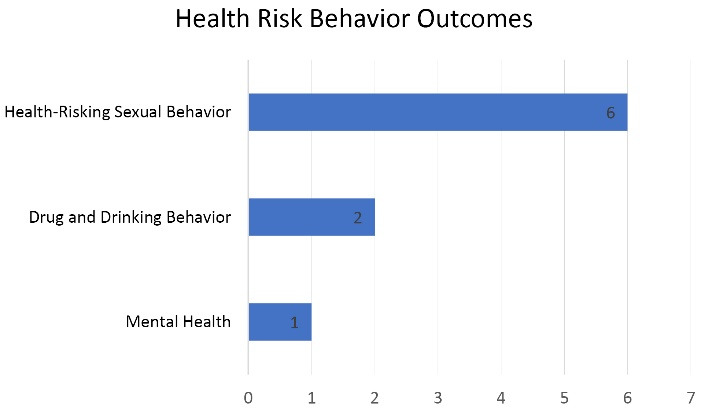
Health risk behavior outcomes

 Community empowerment-based interventions that have been reviewed through the modalities discussed have been shown to affect the outcomes of health risk behaviors. Several risk behaviors that are influenced by these modalities include sexual behavior that is risky to health, drug and alcohol behavior (see Figure 2). Two studies show that community empowerment-based interventions positively reduce health risk behavior,^14,18^ except for the conditional cash payment intervention, which had a little effect in reducing high-risk behavior.^18^

 The community-development intervention on drinking behavior showed a positive impact in reducing regular alcohol consumption.^22^ Intervention in the family setting had a positive influence on mental health by improving family communication.^16^ However, another study showed that the intervention did not significantly affect alcohol use or problem drinking.^22^

## Discussion

 There are three main findings of this study: (1) most reviewed studies show that community development–based interventions are effective in reducing health risk behavior in adolescents; (2) health risk behavior outcomes include health-risking sexual behavior, drug and alcohol use behavior, and negative effects on mental health; and (3) interventions are carried out in schools, places of worship, and communities involving adolescents, educational institutions, health professionals, religious leaders, and family.

 The community can have a significant impact on adolescents’ health-related behaviors. According to the findings of this study, adolescents who receive community development–based interventions are better at reducing health risk behaviors. Community development was also effective in studies of success in increasing adherence to antiretroviral treatment in adolescents with HIV.^23^ Neighborhood and community as well as families also influence cigarette smoking, alcohol and other drug use, and partnered sexual activity.^24^

 The most effective interventions emphasize adolescents active and interactive participation in the community. These methods go beyond simply transmitting information and developing and strengthening life skills.^26^ Life skills enable adolescents to overcome the life challenges effectively, and include solve their problems.^26^ The attainment of life skills promotes mental health, better relationships, and healthier behaviors among adolescents. It also aids in the development of protective factors against health risk behaviors.^25^

 The community development in adolescents can help adolescents overcome challenges in their life, reduce high risk behavior, and improve positive behavior. Hopefully, the systematic structure and delivery of community-development programs will have a significant impact for the adolescents’ health and well-being.

 Nurses can contribute to community development by providing nursing care to adolescents in various settings, including communities, schools, public health, and acute-care clinics. It gives the adolescents many opportunities to improve healthy behavior and reduce health risk behavior.^27^ It is important that nurses deliver evidence-based counseling and services to adolescents and parents in all nursing practice settings to ensure that adolescents have access to health services that can promote healthy behavior, e.g., sexual and reproductive health care.^27^Service learning is a way nurses can learn the skills of counseling and services.^28^

 This study has several limitations. As most studies included were conducted in the USA, it is prudent to exercise caution when extrapolating the findings to other countries. Further, as we only included RCTs, interventions that did not lend themselves to evaluation by that method but may have been effective in reducing health-risking behavior were excluded from our study. Additionally, the authors did not use sources from gray literature and relied solely on research results published in databases.

## Conclusion

 This study shows the importance of community development to overcome adolescents’ health risk behavior. Most studies show that community development–based interventions effectively reduce health risk behavior in adolescents. Interventions based on community development can involve schools, places of worship, general communities, adolescents, educational institutions, health professionals, religious leaders, and families to reduce the multiple health risk behaviors among adolescents.

 The findings of this review can be used in developing programs or interventions based on community development and empowerment to reduce health-risking behavior in adolescents. Few intervention modality studies or RCTs have been undertaken in this area. Further research is needed to examine the effectiveness of community-development interventions in reducing adolescents’ health risk behavior.

## Authors’ contributions

 AY conceived and designed the analysis, collected the data, contributed data or analysis tools, performed the analysis, wrote the paper. SS conceived and designed the analysis, contributed data or analysis tools, performed the analysis. SYR conceived and designed the analysis, contributed data or analysis tools, performed the analysis. NJ contributed data or analysis tools, performed the analysis, wrote the paper.

## Funding

 This study was funded by Internal Research Grants Universitas Padjadjaran, Bandung, Indonesia.

## Ethical approval

 This study used scoping review methods, therefore ethical clearance was not applied.

## Competing interests

 None to declare.

## Disclaimer

 The authors claim that no part of this paper is copied from other sources.
